# Mining Massive E-Health Data Streams for IoMT Enabled Healthcare Systems

**DOI:** 10.3390/s20072131

**Published:** 2020-04-09

**Authors:** Affan Ahmed Toor, Muhammad Usman, Farah Younas, Alvis Cheuk M. Fong, Sajid Ali Khan, Simon Fong

**Affiliations:** 1 Department of Computer Science, Shaheed Zulfikar Ali Bhutto Institute of Science and Technology, Islamabad 44000, Pakistan; affan.toor85@gmail.com (A.A.T.); dr.usman@szabist-isb.edu.pk (M.U.); farah.younas@szabist-isb.edu.pk (F.Y.); 2 Department of Computing, Western Michigan University, Gladstone, MI 49837, USA; 3 Department of Software Engineering, Foundation University Islamabad, Islambad 44000, Pakistan; sajidalibn@gmail.com; 4 Department of Computer and Information Science, University of Macau, Macau 999078, China; ccfong@um.edu.mo

**Keywords:** data stream mining, IoMT, concept drift, class imbalance, machine learning

## Abstract

With the increasing popularity of the Internet-of-Medical-Things (IoMT) and smart devices, huge volumes of data streams have been generated. This study aims to address the concept drift, which is a major challenge in the processing of voluminous data streams. Concept drift refers to overtime change in data distribution. It may occur in the medical domain, for example the medical sensors measuring for general healthcare or rehabilitation, which may switch their roles for ICU emergency operations when required. Detecting concept drifts becomes trickier when the class distributions in data are skewed, which is often true for medical sensors e-health data. Reactive Drift Detection Method (RDDM) is an efficient method for detecting long concepts. However, RDDM has a high error rate, and it does not handle class imbalance. We propose an Enhanced Reactive Drift Detection Method (ERDDM), which systematically generates strategies to handle concept drift with class imbalance in data streams. We conducted experiments to compare ERDDM with three contemporary techniques in terms of prediction error, drift detection delay, latency, and ability to handle data imbalance. The experimentation was done in Massive Online Analysis (MOA) on 48 synthetic datasets customized to possess the capabilities of data streams. ERDDM can handle abrupt and gradual drifts and performs better than all benchmarks in almost all experiments.

## 1. Introduction

Fast pace, continuously flowing data and a very large data size is categorized as a data stream. Storage incapability of accumulating an entire stream in a system and mining fast flowing data poses limitations on the processing capabilities of conventional data mining algorithms, making stream mining a challenging task. Moreover, from such characteristics of streaming data arise the possibility of concept drift, a situation where overtime change in data distribution occurs. 

Transitions in target data distribution can be caused by factors like unorthodox system behavior, data deviation, and changes in environmental conditions. A failure to conform to the Independent and Identically Distributed (IID) principal of probability theory is a big challenge for stream mining, raising the issue of concept drift. When a rapid change occurs in data distribution, it is called abrupt, and when the variation occurs over time, slowly and seamlessly it is called gradual. A variation of gradual concept drift—incremental concept drift—is generated from multiple sources of data streams. In this case, an abrupt or gradual drift occurs repeatedly and forms a pattern, which is called recurring. The Sliding Window Model is probably the most effectively and widely used technique to handle these challenges. Identifying various types of drifts and modifying itself accordingly is imperative for a concept drift detector.

Class Imbalance, a known problem in data mining, arises when the total number of occurrences of one instance is in the majority compared to the other class or classes, causing overfitting problems in the case of a positive class and decreasing the results immensely in case of a negative class. Unlike concept drift, this problem is not limited to streaming data, but found in static data as well. Class imbalance can be found in many domains, such as fraud detection, anomaly detection, medical diagnosis, oil spillage detection, and facial recognition in security, among others. Studies have shown that the problems of concept drift and class imbalance affect the mining capacity and results a great deal. But most of the efforts made to resolve these problems are one dimensional. It is important to handle concept drift and class imbalance simultaneously. The coexistence of concept drift and class imbalance can be found in several real-world examples, such as smart homes, patient activity monitoring devices, IoMT for smart healthcare etc. 

**Figure 1:**
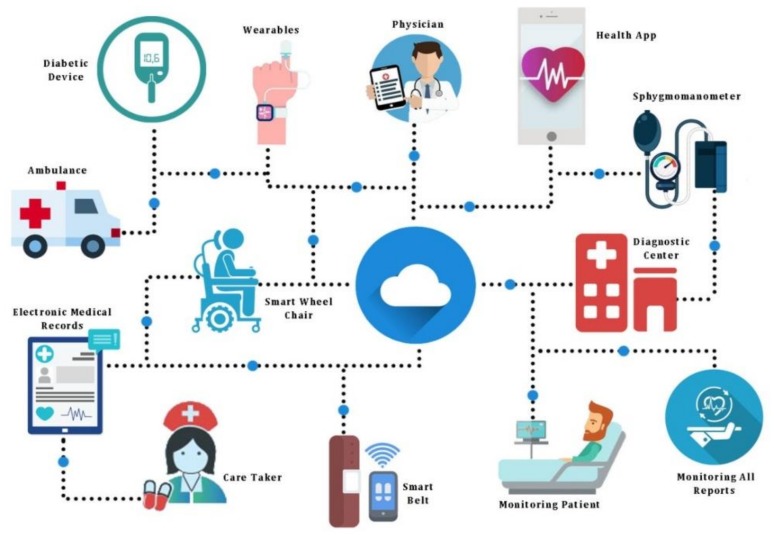
Infrastructure of Internet of Medical Things (IoMT).

The Internet-of-Medical-Things (IoMT) is a collection of smart medical devices and applications, as shown in Figure 1, communicating with each other over a network for smart healthcare. It reduces the burden on healthcare systems by connecting patients to their physicians and allowing the secure transfer of medical data over computer networks. 

As reported by New England College, IoMT includes wearable devices, sensor-enabled hospital beds, infusion pumps, medication-tracking systems, medical supply and equipment inventory tracking, etc. There are smart watches that monitor heart rates and track movement. Similarly, there are contact lenses to read glucose levels, biometric stamps that report the wearer’s vitals, and necklaces that analyze chewing and swallowing—and alert the wearer when they’ve had too many carbohydrates. 

All these devices continuously generate data in the form of a high-speed stream, which needs to be analyzed in real-time. Therefore, in the medical domain, not only is timely analysis important, but also the detection of concept drift also becomes a mandatory task. For example, the medical sensors measuring for general healthcare or rehabilitation may switch their roles for ICU emergency operations when required. However, the occurrence of abnormal patient activity is a typical example of class imbalance. For example, in a typical patient monitoring unit such as an ICU, the medical sensors keep transmitting normal activity data, and very gradually an abnormal activity is been sensed and transmitted by the wearable devices/sensors. This shows the true ratio of imbalance data, which need to be analyzed through cutting edge techniques. 

There are certain challenges when we try to solve concept drift and class imbalance together. First, as we know, it is recommended that the class imbalance removal techniques are applied before passing data to the classifier, so that the classifier does not have to perform any extra computations to cater for class imbalance. Conflict arises when we also have to consider concept drift in the data. An active concept drift approach looks for drift in the incoming data. So, there is a need to configure these steps so that they do not interfere with each other’s processes and results. Reactive Drift Detection Method (RDDM)—which is based on the Drift Detection Method (DDM), a well-known drift detector—claims to be an improved version of DDM, as it detects most drift more rapidly than DDM, and delivers a higher accuracy than DDM in most situations. 

One of the limitations of RDDM is that it takes into account the concept drift of a data stream alone, and does not consider the most commonly occurring problem of class imbalance, not just present in the streaming data, but in the conventional/static data as well. These problems not only co-exist in real life datasets, but also have a negative influence on each other.

This work proposes the Enhanced Reactive Drift Detection Method (ERDDM), a consolidated solution for the problems of class imbalance and concept drift in data streams. ERDDM is an intensified and improved version of RDDM, which tackles the conundrum of treating concept drift along with class imbalance problems. ERDDM is an automated solution that provides an ensemble method to remove class imbalance from a sliding window of live data streams and gets improved results from the balanced data.

In addition, the Synthetic Minority Over-Sampling Technique (SMOTE) approach was adopted to deal with class imbalance, rather than utilizing a cost-function based approach, due to its computational complexity. We claim that ERDDM has enhanced the existing drift detector algorithm to perform better in terms of low prediction errors, high rate of detected drifts, and less processing time.

In this paper, we propose a new concept drift detection method: ERDDM. Our new method efficiently detects the abrupt and gradual concept drifts in large data streams. Moreover, ERDDM has been tested against RDDM, EDDM, and STEPD, using a number of base learners, such as Naïve Bayes, Adaptive Random Forest (ARF), Kappa Updated Ensemble (KUE), Accuracy Weighted Ensemble (AWE), Leverage Bagging, and OzaBagAdwin. Experimental results show that ERDDM is significantly superior to the other three methods in terms of accuracy for detecting imbalanced data streams.

The rest of this article is organized as follows: Section 2 explores the related work of other authors, specifically focusing on different aspects of RDDM; Section 3 describes ERDDM and presents its pseudo-code; Section 4 describes the datasets and configurations settings used for the experiment; Section 5 discusses the results, interprets the identified drifts and analyses their significance, and it also evaluates the results by comparing the prediction error, total detected drifts and runtime; and, finally, Section 6 concludes this article by summarizing the results and final outcome of this study, along with proposing some future directions.

## 2. Related Work

### 2.1. Concept Drift Detection

Researchers have tried to handle concept drift through different approaches over time, which includes sliding window vs. batch processing, fixed length window vs. variable length window, fixed stream speed vs. variable stream speed, labeled vs. unlabeled or partially labeled data points, ignoring the processing results of a window vs. keeping a history of the drift ratio of each window, using a drift alert mechanism vs. maintaining drift logs to be used later, and so on. The whole concept drift detection process has evolved over time by making such choices and enhancing the process incrementally. Researchers have proposed many concept drift detection methods [1,2,3,4,5,6,7,8,9,10,11,12,13,14,15,16] in recent times, which have handled this problem in different ways for different situations.

#### 2.1.1. Abrupt Drift Detection

Some prior work [1,2,3,4] done on detecting concept drift is mainly focused on detecting the Abrupt drift type. OnePassSampler [1] based on Bernstein Bound, makes a single pass through its memory buffer and employs a simple and efficient array structure to maintain data about the current window. Its predecessor, ADWIN2, triggers a reassessment of candidate cut points previously visited with every new data block that arrives. However, OnePassSampler is heavily dependent on the proper configuration of the key parameters, which requires deep knowledge of algorithms. Another method, SEED [2], has separate components for detecting drift and rate of change. SEED creates blocks of data of fixed size and passes on two subsequent blocks to the drift detector. If any deviation is found within these blocks, it is inferred that the drift exists, and these blocks are passed onto the volatility detector, which decides what kind of volatility these blocks may have. SEED assumes that the data distribution will remain the same throughout the stream, which is often not the case in real life data stream. AOGE [3], in combination with Principal Component Analysis (PCA), is a subspace learning technique. PCA analyzes the projection variance of the data and identifies the dispersion and correlation among the data. Meanwhile, AOGE analyzes the projection angle of the data and passes information to PCA. AOGE fails to maintain the integrity of the dataset by inducing a synthetic drift in the data.

#### 2.1.2. Gradual Drift Detection

Work done on gradual concept drift detection includes solutions proposed by Pears et al. [5], Kithulgoda et al. [6], Jaka et al. [7], and Sethi et al. [8]. SeqDrift1 and SeqDrift2 [5] are two methods specifically designed to handle gradual concept drifts. These methods are based on Bernstein Bound, but with a change in its formulation to fix the limitations identified in a prior method: ADWIN. A drawback in SeqDrift1 is that it does not provide the flexibility to assign weightage to a false positive rate or detection delay time. This restrains the usability of this method and makes it harder to generalize it for multipurpose use. A method based on Discrete Fourier Transform (DFT) named Staged Online Learning SOL [6] divides data into two stages, i.e., high and low volatile data. The Incremental Fourier Classifier IFC method is applied to a low volatile stage, so that it incrementally refines the concept changes and provides a base for the high volatile stage. SOL yields good results, but there is a high chance that any features that do appear in the decision trees have a very low or even no impact on the classification process using SOL, which will lead to a misclassification. The Interaction-based Method for Exploration IME-based method [7] uses the model’s explanation paradigm, which gives an insight into its decision-making process. The IME method passes the dissimilarities of the model change to any arbitrary concept change detection method, and observes any change. If a change is identified, then the model is updated. A drawback of IME is that it heavily relies on a large number of parameters, which require prior knowledge to use. The Margin Density Drift Detection (MD3) [8] method monitors the number of samples within the classifier’s uncertain region and detects concept drift according to those samples. It picks the features to be rotated on the basis of an information gain metric, which ensures that the induced drift is not random, but has a high significance for the classification task.

#### 2.1.3. Abrupt and Gradual Drift Detection

Some researchers [9,10,11,12,13,14,15,16] have worked on handling both abrupt and gradual concept drifts. Both EDDM [9] and STEPD [10] have used a statistical testing technique to detect drifts. Their approach is to calculate the difference between two errors, and to then infer whether there is drift in stream or not. However, these algorithms are not scalable to be used for huge data streams. A semi-supervised approach is adopted by [11], which is able to handle (i) drift in the input space due to changing class means, (ii) drift in the class variance, and (iii) drift in the target concept. The main emphasis of this research is to detect streams in partially labeled data, however, the labeling technique used in this method makes the detection process very slow, and at times the drift is detected after 200 or more instances had already been passed undetected. Another study [12] also attempted to handle drifts in partially labeled data by proposing three new methods named Fixed Reference Windows (FRW), Moving Reference Windows (MRW), and Ensemble of Reference Windows (ERW). These methods performed better than [11], however this solution only works for two-class data streams, which limits its practical usage.

Another method, k-Nearest Neighbor-based space-Partitioning Schema NNPS [13], caters for drift detection by finding the regional density-based similarity measurement, which can cause the concept drifts to be sensitive to local or global distribution changes. NNPS can accommodate both one dimensional and high dimensional data in lieu of a distribution driven concept drift detection. However, there is less evidence that NNPS will be scalable for varying window sizes and stream speeds. Another method, the Kullback-Leibler Divergence (KLD) [14] measure, is used, which compares different components of the ensemble and decides which component is best for the current data points. 

The proposed method, Automatically Adjusting Size of Ensemble Algorithm with Temporal Changes (ASE-TC), works by comparing the results of the weakest classifier in the ensemble for a given data chunk. The KLD value is computed for that classifier, and if it is greater than the user-defined threshold, then that classifier is used in the ensemble for next iteration. KLD works well within a controlled environment where the expected drift type is known and the KLD value is also predefined. Another ensemble-based technique known as Drift Detection Ensemble DDE [15], is backed up by multiple weighting functions to calculate the classification results. All the detectors within DDE share the same centralized information of data points. However, only one detector will be active and working at a time. DDE performs better with synthetic datasets, but fails to deliver for real-world datasets. A similar ensemble-based method called Hoeffding’s bound Drift Detection Method HDDM [16], is based on Hoeffding’s Bounds, and calculates the mean estimation of the classification results to detect both types of drifts. HDDM takes an iterative approach by running an algorithm 30 times before inferring the final results—for a real-world high-speed data stream this could be a very complex and time-consuming process, which may result in slow performance.

### 2.2. Class Imbalance

Most of the work [17,18,19,20,21,22,23] done in removing class imbalance from data streams is based on ensemble-based techniques. Oversampling based Online Bagging (OOB) and Undersampling based Online Bagging (UOB) [17] can solve the online class imbalance problem for a two-class dataset and are capable of monitoring changes in the class imbalance and adapting to the changing class distribution without outside intervention. However, the results indicate that this solution is not suitable for high dimensional large data streams. Miguéis et al. [18] have targeted the problem of predicting customers’ response to direct telemarketing campaigns in a banking context. They have used an external approach, which applies resampling techniques to data before applying a classifier. The RandomForests classifier for prediction and the EasyEnsemble resampling technique for handling class imbalance problems are suggested as an efficient combination for the classification of a direct marketing dataset with class imbalances. Experiment results show good ROC and AUC, however, the proposed ensemble is tailored for telemarketing data and it is not scalable to be used for other industries. 

Chaoliang et al. [19] have done work on the class imbalance problem in the context of Twitter spam detection. Due to class imbalance, the spam detectors have not been able to perform up to their potential. A novel fuzzy-based ensemble is proposed and compared with other algorithms that handle class imbalance. When compared with algorithms like SVM, KNN, Naive Bayes, C4.5, NB, etc., the proposed ensemble showed promising results in almost all experiments on both test datasets as well as the Twitter dataset. The results, however, show that with increased class imbalance rate, the performance of proposed ensemble decreases. Another study [20] discusses the class imbalance problem in the context of an unsupervised change detection analysis of Very High Resolution VHR remote sensing imagery, and provides s systematic description of the nature and effects of imbalanced classes in this exemplary application setting. The proposed methodology serves as a diagnostic analysis framework to evaluate transferability with respect to an arbitrary distribution of classes in any two-class classification problem. An object-based feature set controls the absolute accuracy of change detection, while the utilized clustering algorithm possesses the highest influence on the classification performance. But on the other hand, the selection of these object-based features is done by extracting the color schemes from the sensor images. Factors like sensor camera quality, complex structures of buildings, ambiguous or undecidable terrain, etc. can easily influence the extraction of the wrong object-based features, which will eventually affect the overall process. 

Another study [21] on class imbalance reduces the gap between the actual class imbalance and the amount identified by the classifier, by adopting a different perspective on the matter. A method is proposed, which shortens the class distributions and calls it Imbalance Degree (ID). This single value consists of binary and multi-class classification problems, and represents the difference between a purely balanced distribution and the studied unbalanced problem. The results conclude that the proposed ID methodology is able to cope with class imbalance more efficiently, even on an individual instance level. However, there are a limited number of basic distance/similarity functions used for this study. For better results the experiments should be conducted on more advanced distance/similarity functions. Another study [22] does not just rely on one sampling method or one classifier to detect spam for imbalanced class data, instead it applies a number of different sampling techniques and a variety of classifiers, and then uses a voting mechanism to select the best results. A three-step process is introduced to come up with an efficient Twitter spam detection methodology. However, the experimentation results show that when the imbalance rate increases, the performance of the proposed method decreases.

According to [23], in the field of botnet detection, where bots or the devices owned by common users, bots are hijacked by a bot master, who starts to control the bots anonymously and seamlessly, in a way that mixes the normal behavior of the bots with malicious behavior. Since the ratio of these bot activities is always very small, as low as 0.1%, it creates the problem of class imbalance, and requires a proper method to remove class imbalance and detect botnets. A new framework is proposed in this study, which is based on the Genetic Programming (GP)GP under label budget. It requires limited level human intervention to train the GP classifier, which then works indecently to classify bots and normal data. The label budget policy is specified for this framework, which decides, upon data arrival, how many instances needs label request.

A strong point of the proposed framework is the implementation of two-dimensional sampling and archiving policies, i.e., uniform and biased. First, the uniform sampling is applied, and then, on the basis of uniform sampling results, biased sampling is applied. This is done before the data is passed onto the GP classifier. Later, the archiving policy is implemented, which decides the type of archiving on basis of GP classifier’s results.

### 2.3. Concept Drift with Class Imbalance

In an earlier study on the combined solution of concept drift and class imbalance, Ditzler et al. [24] designed an ensemble framework, which solves the concept drift and class imbalance problems, regardless of drift type and imbalance ratio, and provides a stability-plasticity characteristic to the stream. Two separate techniques are proposed, which are based on different mechanisms, in order to solve these problems from multiple perspectives. Learn++.CDE is based on SMOTE, whereas Learn++.NIE is based on bagging. Unlike previous studies, the proposed methods do not have any selection criteria, for instance filtering; instead, they use all instances from the batch and try to learn from most of the data instances. Learn ++.CDE assigns penalty weights to the miss classifications on basis of drift frequency, such that in the case of no drift, the weight is very high, and in case of high drift, the weight is very low. These penalty weights are used for relearning of the model. Learn ++.NIE refrains from oversampling; instead, it uses the bagging technique and avoids adding synthetic data. According to the authors, the proposed approaches require extra computational power because of the weighting mechanism. This factor will be crucial in case of a real-world high-speed data stream with highly skewed distributions of data.

In [25], Mirza et al. proposed an Ensemble of Subset Online Sequential-Extreme Learning Machine ESOS-ELM ensemble that processes the minority class instance with specified list of classifiers, and in the case of a majority class, a round robin routine is used. ESOS-ELM is primarily targeted to work with both class imbalance and concept drift, but it is also capable of handling concept drift without the presence of class imbalance. Furthermore, it is designed to handle concept drift and class imbalance in both stationary and non-stationary environments. The proposed ensemble maintains two lists of classifiers, one having a number of minority class samples classified by model and the other having a number of majority class samples. These lists are sorted in ascending order, and when the class imbalance ratio lies within the first list, the instance is processed by the top classifiers in that list and vice versa. This way, the proposed method decides which ensemble is best suited for the incoming instances, on basis of an imbalance ratio. However, there are a few shortcomings found in the model. First, there is a limitation in the proposed ensemble, in that it is not able to add new classes to its learning in the middle of the process. If a new class arrives, then the model has to relearn everything from the start. Secondly, the proposed ensemble works with an initializing of the hidden parameters and neurons, which remain the same throughout the process. This could be improved by accommodating the varying neurons and parameters upon the detection of certain types of drifts. 

In a similar study done by Ren et al. [26], authors proposed the Gradual Resampling Ensemble (GRE) method, which combines the qualities of chunk-based ensembles and online ensembles. It is capable of detecting changing concepts quickly, due to its incremental oversampling of a minority class, which enables it to predict data distributions. Unlike most similar solutions, the data difficulty factor of minority instances is not ignored, due to its disjuncts-based clustering mechanism. GRE performs density-based clustering on the data to exclude outliers, and these clusters are based on minority and majority classes. Analysis done on Massive Online Analysis (MOA) shows that the proposed model is not affected by the varying chunk sizes, hence, it performs equally well on small and large chunk sizes. However, in order to restrict memory usage, the proposed method applies selective resampling. This means that all data instances are not used for processing. Studies show that selective resampling does not represent the true nature of the data and, especially in case of drifts, either the drift is totally removed, or its behavior is changed. 

Another recent study by Wang et al. [27] gives an insight into how to select an appropriate drift detector which is sensitive to class imbalance as well. According to the findings of this paper, the effect of class imbalance is more lethal when it is in the presence of concept drift. This is because the class imbalance makes it harder for models to detect concept drift, and the drift detector gives false drift alarms. Furthermore, the gradual concept drift, referred to as real concept drift, is the most difficult type of drift to catch, and it is also highly affected by the class imbalance problem. On the other hand, the abrupt concept drift is immune to the class imbalance problem, and hence, it can be treated with any model which ignores the class imbalance. The authors highlight another aspect of concept drift, which is that not all types of drifts change the decision boundary. So those models which reset their setting in case of any drift occurrence basically waste resources and overdo the processing. A model should be reset on the basis of certain drift types, and their influence on the decision boundary.

In a recent study, a Kappa Updated Ensemble (KUE) classification algorithm was proposed by Cano and Krawczyk [28] for drifting data streams. The proposed ensemble-based algorithm uses the Kappa measure to select and assign weights to the base classifiers. They introduced diversification techniques by combing online bagging with random feature subspace for the base learners. With this approach, the class imbalance issue for the streaming problem has been tackled by maintaining the robustness to drift. Experimental results proved that their proposed algorithm for drifting stream has superiority over the existing state-of-the-art ensemble methods. The same authors have also proposed an evolving rule-based classifier named ERulesDS [29] for drifting streams. Genetic programming was exploited for the adoption of these rules. High scalability was achieved by implementing the proposed rule-based classifier on GPU based machines. Both the aforementioned approaches have obvious advantages over the existing classifiers used as base learners in most of the stream mining tasks. However, there is a need to test and compare these new ensemble-based and rule-based classifiers with the current concept drift detection methods such as RDDM, STEPD, and EDDM. Unlike the work reported in [28,29], our study focuses on proposing a new and efficient concept drift detection algorithm by comparing it with the existing algorithm in terms of accuracy. However, in this study, we focus on the comparison of drift detectors, instead of scalable implementation and automatic selection of base classifiers. The powerful base learners i.e., KUE and ERuleDS, will augment our work, as automated selection base learners can also enhance the power of drift detectors.

## 3. Enhanced Reactive Drift Detection Method (EDDM)

This section provides technical insights to ERDDM, our proposed drift detection method that provides the concerted solution for abrupt and gradual concept drifts, as well as class imbalance in the data stream. This includes the implementation detail of ERDDM in MOA, and also describes the deficiencies highlighted in RDDM, which our proposed method aims to resolve.

The main idea behind ERDDM is to increase the performance by decreasing the error rate in the drift detection process in RDDM. The RDDM model was based on two basic assumptions, i.e., first, when there are large numbers of data points belonging to same concept, the mean error rate is not affected until after a large number of prediction errors are reported. This causes a delay in drift detection and ultimately results in decrease of classification accuracy. Second, both DDM and RDDM use two levels of notification to pass on the information to the classifier of a drift in the data. The first level is the warning, i.e., when a potential concept drift is identified, then a warning is generated, and the preceding instances are monitored for the continuation of the drift. In case the drift continues, after a specified threshold the alert is generated, and the classifier is notified of the drift. At this point, however, the type of drift is not determined, because it could take few windows to clarify whether the drift is abrupt or gradual. DDM had a weakness to stay at warning level for longer period of time. 

To handle these problems, a few design decisions were made by RDDM, such as re-calculating the DDM parameters with a smaller number of instances within a window to determine the existence of drift and generate an alert in time. So, what RDDM does is that it reduces the amount of data points in a concept on every iteration, so a large concept is divided into small parts, which makes it easy to process for drift detection. Another decision made by RDDM is to avoid re-calculating the DDM parameters once the warning level is generated. The reason behind this is that recalculating during the warning level will make the algorithm work slowly, and there is also a chance of over-calculation in the case of a long concept drift. As a workaround, a threshold of instances is set by RDDM before the warning level is converted into alert level. Until this threshold is not achieved, the re-calculation of the DDM parameters is not done. There are two possibilities in this scenario: first, if the detector issues a warning and a certain amount of data points are processed and the warning is still on, it means that the drift, although not yet confirmed, most probably has already started, so in that case, the warning level is increased to notify the classifier to adjust itself. Second, if the drift occurs during the warning level, then it means that most probably the starting point of the drift was the same instance when the warning was generated. So RDDM starts to re-calculate the parameters.

RDDM added three new parameters to the list of DDM parameters in order to implement the new logic. A total of six parameters are used by RDDM, which are n: minimum number of instances before monitoring changes; αw: warning level; αd: drift level, max: maximum size of concept, min: minimum size of stable concept and warnLimit: warning limit of instance. The default values of these parameters set by RDDM are n = 129, αw = 1.773, αd = 2.258, max = 40000, min = 7000 and warnLimit = 1400, respectively. We have kept these six parameters and their default values in our proposed algorithm, because some experimentation showed that these default values work well with our changes too. Also, comparing ERDDM with RDDM will be more relevant if both are tested on the same parameters.

### 3.1. Page Hinkley Test

The core change introduced in the RDDM by this study is the replacement of the concept drift detection formula used by RDDM with the Page Hinkley (PH) Test. The Page Hinkley Test is widely used as the method to detect change in Gaussian Signals. It has a variety of implementations, and researchers have used PH for concept drift detection as well. We believe that the error rate in drift detection can be reduced by applying the PH test at the warning level. The formula for the PH test is as follows:

(1) $$m_{t}=\overset{n}{\underset{t=1}{\sum }}\left(x_{t}-\underline{x\;}\right)+\alpha$$

- where $\underline{x}=\frac{1}{t}\sum_{l=1}^{t}\left(x_{l}\right)$
- α corresponds to the magnitude of changes that are allowed
- The minimum value of $m_{t\;}$ is computed as: $M_{t}=\left(m_{t}\;;\;t=1,\;\ldots ,\;T\right).$

This test basically monitors the difference between $M_{t}$ and $m_{t}$ by:

(2) $$PH_{t}=M_{t}-m_{t}$$

When this difference is greater than the threshold (λ) then it is declared that there is a change in distribution, and the algorithm generates a warning to notify the classifier about the drift.

### 3.2. Core Algorithm

Algorithm 1 shows the pseudo code of ERDDM. The code is implemented in Java language and runs on the Massive Online Analysis (MOA) tool. The algorithm starts with initializing the variables, e.g., rddmDrift and ddmDrift are set to false, a new byte array of storedPredictions is initialized, numInstConcept and numWarning is set to 0, and m_t_array is initialized, which will hold the PH-related m_t values. Our main focus is on the part where change is calculated and compared with the threshold. We have introduced the Page Hinkley Test for the drift detection steps, and these steps can be found from line 17–19 in the algorithm. First, *m_t* is calculated using the aforementioned PH formula. Then the result of *m_t* is appended in an array. This is required to maintain the history of the *m_t* values within the window, so that *M_t* can be calculated by picking the minimum value from the *m_t* array. Lines 23 and 27 have the main conditions to check the threshold values of M_t and m_t. On basis of these conditions the drift, and warning levels are set on line 24–26 and 28–33.

### 3.3. Combining Class Imbalance Solution with ERDDM

The process starts with an input data stream. A sliding window model is used for accepting and reading the stream. The window size is set to 1000 data points, based on stream speed and available memory on the test machine. Data points of the current window are passed onto resampling ensemble. This module is responsible for handling class imbalance. Class imbalance is removed by using the SMOTE technique, which synthetically generates the data points of the minority class. After resampling, the data is passed onto the drift detector. The reason resampling is done before drift detection is that resampling changes the demographic of the data, so there is a high chance that if the drift detection is done first, then the nature of the drift will be changed after resampling, which will affect the results. As discussed in literature review, there are two approaches to detect drift, i.e., active and passive. Our proposed model followed the active approach, which means the drift is detected in the incoming data, because the computational cost and complexity of a changing model is infeasible for passive approach. Drift detector is designed to handle two types of drifts, i.e., abrupt and gradual. A history of drift is maintained in order to detect gradual and recurring drifts, since such kinds of drifts are spanned across multiple windows. An archiving policy is devised for drift history maintenance, because only a limited space can be consumed. Such a policy can be based on removing old drift history after a certain time, or by calculating the weightage of drift information and only keeping those which have weightage above threshold level. Finally, the processed data points are forwarded to the classifier, which makes adjustments in case of drift, and then the classification results are yielded.
**Algorithm 1.** Enhanced Reactive Drift Detection Method**Input:** stream, n, αw, αd, max, min, warnLimit storedPredictions ← NEW byte [min] reset m_n,m_p,m_s,m_pmin,m_smin,m_psmin rddmDrift ← ddmDrift ← false numInstConcept ← numWarnings ← 0 m_t_array ← [ ] Output: 1. **foreach** instance **in** stream **do** 2.   pred prediction (instance) 3.  **If** rddmDrift **Then** 4.   reset m_n,m_p,m_s,m_pmin,m_smin,m_psmin 5.   Calculate DDM statistics using the element of storedPrediction 6.   rddmDrift ← ddmDrift ← false 7.   numInstConcept ← numWarning ← 0 8.  **End If** 9.  Inserts pred into array storedPredictions forgetting oldest value if it is already full 10. m_p ← m_p + (pred – m_p)/m_n 11. m_s ← sqrt (m p × (1 – m_p)/m_n) 12. m_n ← m_n + 1 13. numInstConcept ← numInstConcept + 1 14. warningLevel ← false 15. **If** numInstConcept _ n Then 16.   x_avg ← (1/m_n) x m_p 17.  m_t ← (m_s - x_avg) + n 18.  m_t_array.add(m_t) 19.  M_t = min (m_t_array) 20.  **If** M_t – m_t < m_psmin **Then** **21.**   m_pmin ← m_p; m_smin ← m_s **22**.   m_psmin m_p + m_s **End If** 23. **If** M_t – m_t > m_pmin + αd × m_smin **Then** **24**.  rddmDrift ← ddmDrift ← true 25.  **If** numWarnings ← 0 **Then** **26.**   storedPredictions ← pred 27.   **End If** 28**. else If** M_t – m_t > m_pmin +αw x s_min **Then** 29.  **If** numWarning >= warnLimit **Then** 30**.**   rddmDrift ← ddmDrift ← true **31.**   storedPredictions ← pred 32.  **else** **33.**   warningLevel ← true 34.   numWarnings ← numWarnings + 1 35.  **End If** 36. **else** numWarnings ← 0 37. **End If** 38. **If** numInstConcept >= max and not warningLevel Then 39**.**  rddmDrift ← true 40. **End If** 41. **End For**

## 4. Experimental Setup

All experiments have been performed on a Core i7 Apple MacBook Pro with 16GB RAM and a 500GB storage disk. The experiments were performed using the Massive Online Analysis software developed by University of Waikato, New Zealand. An ensemble of class imbalance removal filters and techniques was used to remove class imbalance, and then an enhanced version of the drift detection algorithm was used for the detection of different types of concept drifts. The following subsections explain the dataset details and the steps performed in the experimental study.

### 4.1. Dataset Details

For experimental study, synthetic benchmark datasets are used, which are very common in concept drift research. There is a total of 42 synthetic datasets used in this experimental study. These datasets are chosen because of their relevance to the drift detection process. It is important to mention here that, for the entire synthetically-generated data streams, different drift sequences were used. For example, for all 50K datasets, we used an interval of 1000 instances to induce drift. Similarly, for 100K datasets, an interval of 5000 instances was used. The detail of these datasets is as follows:

- The Agarwal Generator is a widely used dataset. It contains the hypothetical data of people applying for a loan. It is a two-class dataset, with class A representing the approval of loan and class B representing rejection of loan. The attributes of this dataset are Salary, Age, Commission, Education Level, Value of the House, ZIP Code, etc.
- The LED generator dataset is based on the problem of displaying a specific digit on a seven-segment LED display. It includes 105 noises, which gives an equal amount of probability to each digit. The dataset comprises 24 attributes with both numeric and nominal attributes.
- The RandomTree generator is another synthetic dataset generator which creates the dataset consisting of randomly generated tree branches. The dataset comprises five nominal and four numeric attributes.
- A mixed generator is a concept drift dataset generator, which makes use of the combination of two numeric and two Boolean attributes to induce drift in data. The formula used to create values of these attributes is $\sin\left(3\pi x\right)$.

### 4.2. Comparison of Datasets

A comparison of balanced datasets that are used in the experimental study is given in Table 1, to understand the purpose and usage of each dataset. These datasets are chosen on basis of their repetitive usage in the studies done by many researchers for various research domains.

A similar comparison of imbalanced datasets is given in Table 2. These datasets are generated from the same generators that are used for balanced datasets. The class imbalance property is added using a stratified sampling technique. The imbalance ratio is 90:10, which is most commonly used for such experiments.

## 5. Experimental Result and Analysis

### 5.1. Synthetic Datasets

The experimental study is performed on the synthetic datasets with the following objectives to test certain aspects of the proposed algorithm:

- A comparison of performance of four algorithms: ERDDM, RDDM, EDDM, and STEPD, is analyzed to check the impact of drift detection on the classification model.
- This empirical study of balanced datasets is performed on four synthetic datasets with six different data sizes each, i.e., Agarwal, LED, Mixed and RandomTree datasets with 50,000, 100,000, 500,000, 1,000,000, 2,000,000 and 3,000,000, respectively.
- The experiment on imbalanced data is performed on three synthetic datasets with three sizes each, i.e., Agarwal, Mixed and RandomTree datasets with 50,000, 100,000, and 150,000 sizes.
- Prediction error is the probability an event will happen. It is a calculated value of probability of each possible outcome to occur after the classification is performed. If the prediction error is zero or close to zero, then it can be deduced that the outcome is the expected result. The more it deviates from zero, the result becomes incorrect. Table 3 and Table 4 show the prediction error values of both balanced and imbalanced datasets, respectively.
- Analysis of the time taken to detect drift by four algorithms is performed. This is important because the efficiency of an algorithm, especially a stream-mining algorithm, is highly dependent on how much time it takes to complete its operations. High speed data streams do not give enough time for models to perform operations, so if an algorithm is giving very high accuracy but taking more time than available, then the efficiency of that algorithm becomes useless. Table 5 and Table 6 show the result of time acquired by the drift detector algorithms.
- Drift detection delay is a critical measure in concept drift detection. It is the number of instances used by detectors to realize that the concept drift has started in the data stream. Ideally, it should be within a window, and not more than a few data points, otherwise the model accuracy starts to drop. Most of the researchers used this measure to show the efficiency of their algorithm. Table 7 and Table 8 represent the average drift detection delay for the two sets of synthetic datasets.
- Another empirical study performed was the comparison of the total number of drifts detected by each algorithm. This experiment is important because of two aspects. First, if the detector is detecting more drifts than are actually present in the data, then it means that the detector’s setting is making it sensitive and it needs tweaking. Similarly, if a detector detects fewer drifts than the actual number, then it means the detector is unable to detect drifts of various natures. Table 9 and Table 10 show the results of the total number of drifts detected by the algorithms for both balanced and imbalanced sets.

### 5.2. Real-World Datasets

In this section, experiments have been performed on two commonly used real-world datasets, namely electricity and intrusion ietection. The electricity dataset has been taken from the MOA data stream repository. This dataset has been described by M. Harries and analyzed by Gama, and it was collected from the Australian New South Wales Electricity Market. The intrusion detection dataset has been taken from the UCI KDD Cup repository. The intrusion detector learning task is to build a predictive model capable of distinguishing between “bad” connections, called intrusions or attacks, and “good” normal connections. The experiments performed on the real-word datasets evaluates following aspects of the proposed algorithm:

- A comparative analysis of three benchmark algorithms, i.e., RDDM, EDDM, and STEPD, is performed with proposed ERDDM algorithm.
- The experiments utilize state-of-the-art classifiers, such as AWE, ARF, OzaBagADWIN, and Leveraging Bagging, to analyze the results.
- The results are evaluated on the basis of two measures: accuracy and mean evaluation time. Table 11 and Table 12 show the results obtained through our experimentation. The results obtained through our experimentation are shown in Table 11 and Table 12.

## 6. Discussions

The results, as seen from Table 3, Table 4, Table 5, Table 6, Table 7, Table 8, Table 9, Table 10, Table 11 and Table 12, show various trends from different perspectives, and the following sub-sections elaborate these results further to infer meaningful conclusions. Since multiple measures are used to evaluate the performance of both synthetic and real-world datasets, we explain the results in the context of each measure accordingly. It is worth mentioning that for our experimentation, different stream speeds were tested i.e., 1000 instances/s, 1500 instances/s, and 2000 instances/s. However, we did not find any noticeable difference in our results, and that is why the results with 1000 instances per second are discussed in the following section.

### 6.1. Prediction Error Analysis of Synthetic Datasets

**Figure 2:**
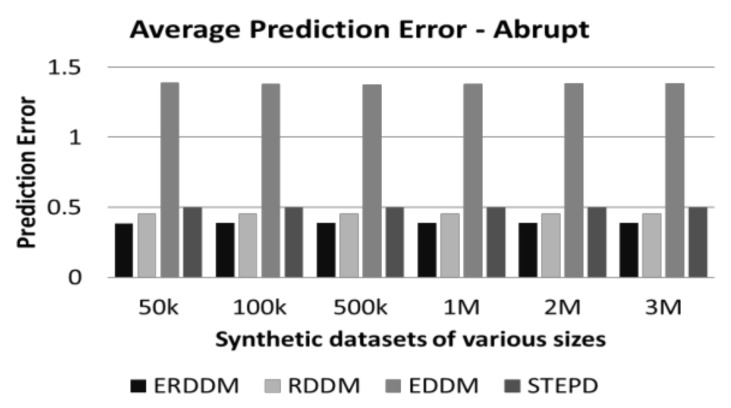
Average prediction error in datasets with abrupt drift.

**Figure 3:**
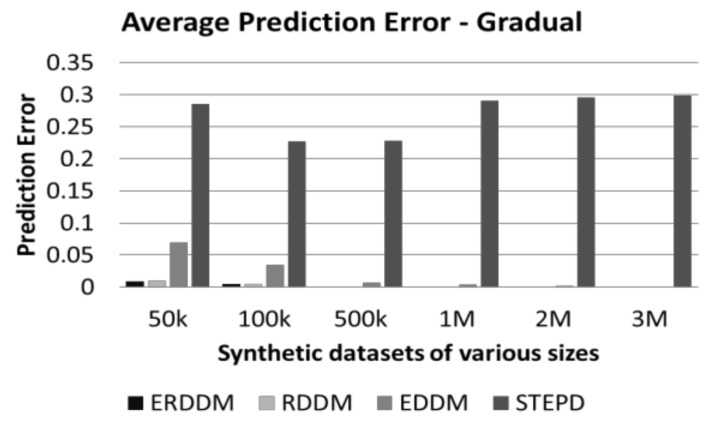
Average prediction error in datasets with gradual drift.

Every concept drift directly affects the classification results and worsens the accuracy of the classification model, so if we keep a check on the error rate of the classification accuracy, then we can detect if the numbers deviate from the normal, and deduce that there is drift in the concept. Visualization of the performance of four algorithms, i.e., ERDDM, RDDM, EDDM, and STEPD, in terms of prediction error is given in Figure 2 and Figure 3.

It can be seen that ERDDM has outperformed all other algorithms and has the least prediction error for all datasets of all sizes. We believe the reason for this is the fact that we have incorporated the Page Hinkley Test, which is used specifically for calculating error rates in data. It can also be seen that ERDDM is scalable, which means with the increase in stream size, the error rate of ERDDM decreases. In comparison, the error rate of STEPD increases with the increase of stream size.

### 6.2. Drift Detection Delay Analysis of Synthteic Datasets

**Figure 4:**
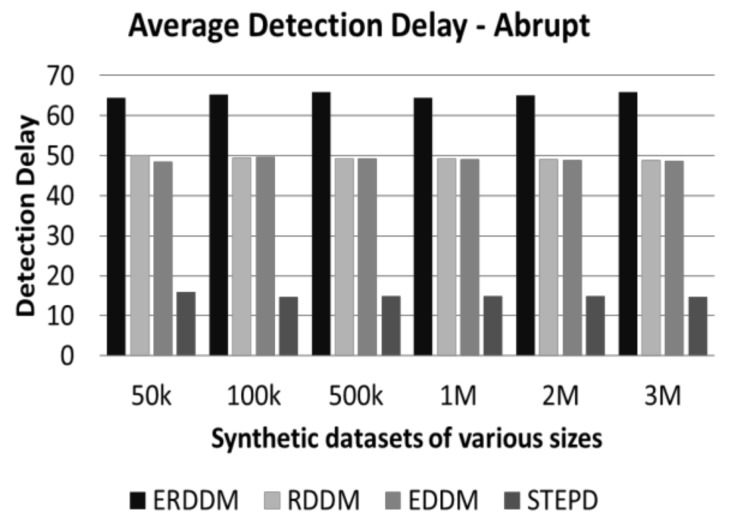
Average detection delay in datasets with abrupt drift.

**Figure 5:**
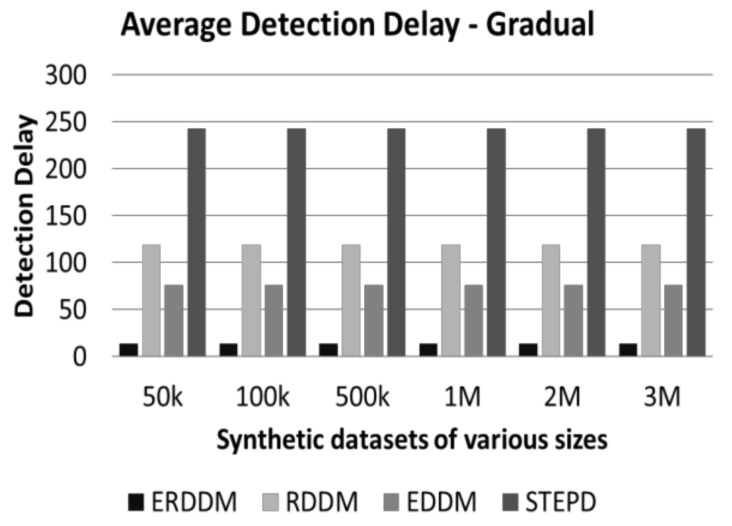
Average detection delay in datasets with gradual drift.

This test is very important in concept drift detection, because the sooner the drift is detected, the sooner the model can relearn and is able to handle the forthcoming data points. Otherwise, with passing of every data point, the accuracy of classifier decreases, and the results worsen over time. Figure 4 and Figure 5 represent the performance of drift detection delay for all algorithms.

**Figure 6:**
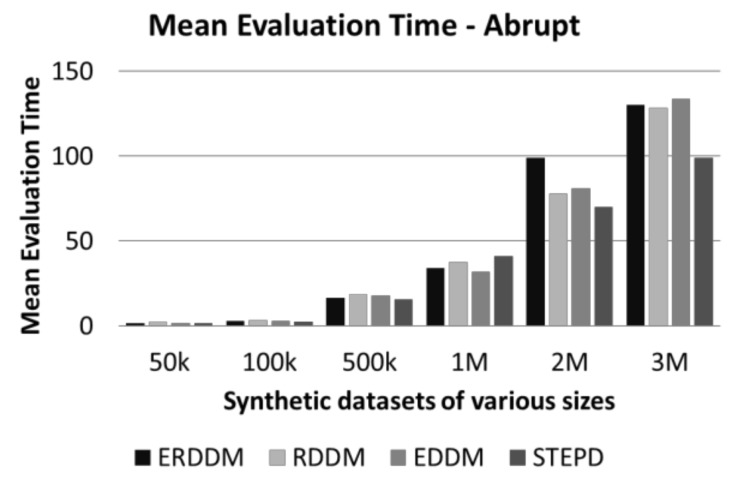
Mean evaluation time of datasets with abrupt drift.

**Figure 7:**
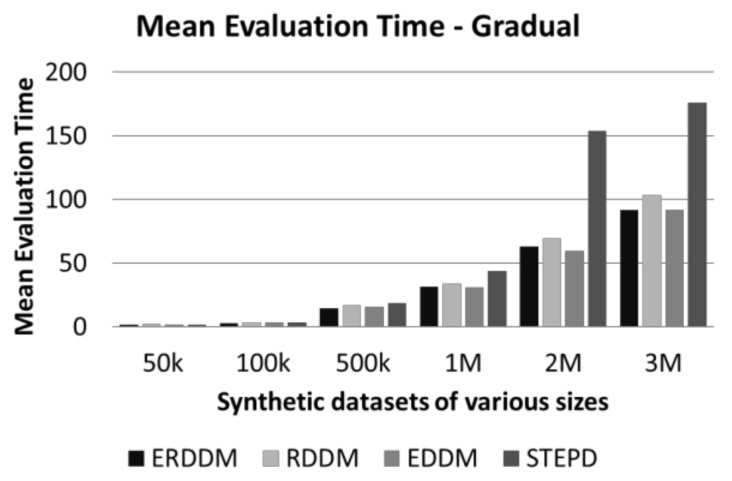
Mean evaluation time of datasets with gradual drift.

We can see from Figure 6 and Figure 7 that not only is the detection delay for ERDDM minimum, but also there is a deviation of only 10 points in drift detection delay for all sizes of datasets. In contrast, RDDM deviates for 20 points, EDDM for 30 points, and STEPD for almost 50 points.

### 6.3. Time Analysis of synthetic and real-world datasets

Time analysis is another measure which we have used to analyze the performance of ERDDM. The amount of time an algorithm takes to process data points in a sliding window is very important, because, in fast flowing data streams, there is not much time to process data. If the algorithm is slow, or responds late, then there is a high chance that it will miss data points for the next window. This becomes a bigger problem in the case of concept drift detect, as, if the missed data points contain the start of a drift, then the drift detector will miss them, and it will result in detection delay and ultimately a degradation in classification accuracy. Figure 6 and Figure 7 show a comparison of the average time taken for all algorithms to detect drift.

As can be seen, there is not much difference in the average times of ERDDM and RDDM, which is understandable because ERDDM is actually an enhancement of RDDM, hence, their algorithm complexity and run time are same. However, if we compare these times with the EDDM algorithm, then a clear difference can be seen, as it takes more time to process. However, STEPD shows less time taken to detect concept drift, but if we compare that with the detection delay and error rate, it shows that STEPD might be taking less time, but not doing much in terms of detection accuracy.

The experiment results in Table 11 and Table 12 show the mean evaluation time for the two real-world datasets. It can be seen from the tables that for the electricity dataset, which has a fewer number of instances, both RDDM and ERDDM take more time compared to the other two drift detectors, regardless of the base classifiers. The same trend has been observed for the larger dataset of intrusion detection. However, it is obvious that our proposed drift detector takes almost the same amount of time compared to its predecessor algorithm, RDDM. 

### 6.4. Drift Count Analysis

**Figure 8:**
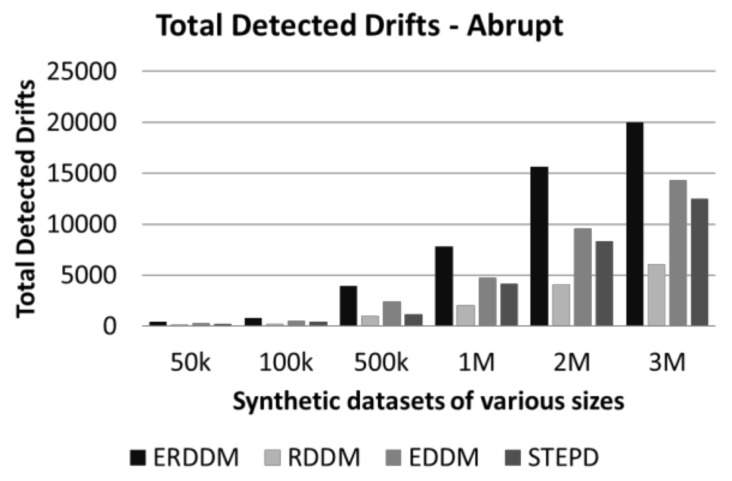
Detected drifts in datasets with abrupt drift.

**Figure 9:**
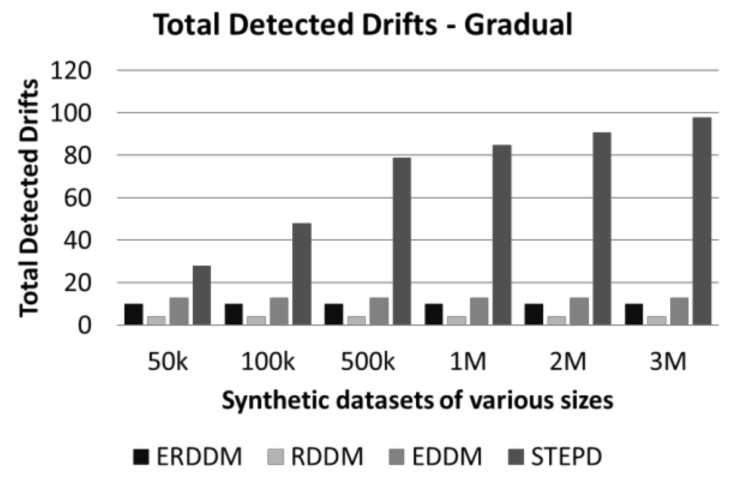
Detected drifts in datasets with gradual drift.

Drift count analysis is another aspect of evaluating the drift detection methods. It is important because if a drift detector is accurate, then it will identify only true drifts in data and ignore the warnings and false alarms. It is to be established that all fluctuation in data are not drifts, as minor changes in classification are normal, and the classifier is trained to handle them. But a drift is identified when these fluctuations exceed the threshold level. If a drift detector is too sensitive, then it might wrongly detect minor fluctuations as drift. But also, if a drift detector is not smart enough, it will ignore the drifts, and result in a lower prediction accuracy and a higher prediction error. The comparison of drift detection count of four algorithms is given in Figure 8 and Figure 9.

### 6.5. Class Imbalance Analysis

Two types of experiments are performed on synthetic datasets, i.e., with class imbalance and without class imbalance. A common trend that can be seen in most of the results is that the prediction error tends to increase in the case of imbalanced data. This shows the impact of the class imbalance problem on the classifier results, and also on the drift detection process. This experiment also proves that the prediction error achieved with imbalanced data is close to the prediction error with balanced data. Since we know that even imbalanced data gives better or equal results, the true positive rate and false positive rate in imbalanced data is always the deciding factor in assuming the accuracy of results. Our proposed algorithm has achieved almost the same results with balanced data. These good results can be comparable to other drift detectors as well.

### 6.6. Prediction Accuracy for Real-World dataset

It can be seen from the results presented in Table 11 and Table 12 that ERDDM has outperformed all other algorithms and has the highest for all datasets of all sizes. We believe the reason for this is the fact that we have incorporated the Page Hinkley Test, which is used specifically for calculating error rates in data. It can also be seen that ERDDM is scalable, which means with the increase in stream size, the prediction accuracy of ERDDM increases. In comparison, the accuracy of STEPD decreases with the increase of stream size.

## 7. Conclusions and Future Work

This article has proposed ERDDM, a new method for detecting concept drifts in data streams generated by sensors and smart devices. ERDDM enhances RDDM by decreasing its error rate and by incorporating the Page Hinkley Test method into the RDDM algorithm. ERDDM possesses the features of RDDM, such as detecting long concepts, keeping history of concepts and updating them over time, periodically recalculating the statistics for drift and warning levels, and dealing with loss of sensitivity in its predecessor. Moreover, ERDDM also incorporates the solution for the class imbalance problem, which is quite common in sensor data of certain domains. Studies show that concept drift and class imbalance often co-exist in data streams, and if both problems are not solved together, then they affect the prediction result to a great deal. Class imbalance is removed using the SMOTE method, which synthetically oversamples the minority class. For evaluation of the proposed method, 48 synthetic datasets are used, which represent patterns of real-world sensor data. ERDDM is compared with its predecessor RDDM, as well as EDDM and STEPD, which are also drift detection methods from prior studies. The synthetic datasets contain both abrupt and gradual drifts, hence, the evaluation is done separately for both types of drifts. The experiment results show that ERDDM outperforms other methods in prediction error, detected drifts, mean evaluation time, and drift detection delay. It also shows scalability with both small and large size of datasets. 

Possible future directions from here could be to detect drift types other than abrupt and gradual, such as incremental and recurring. However, the challenge is that acquiring such data streams that have these rare types of drifts is very hard. Generalizing an algorithm to handle all types of drift could also be a very complex task. Another future direction could be to handle partially or fully unlabeled data streams in conjunction with a concept drift and a class imbalance solution. This study can also be used for other application areas that generate data streams with both concept drift and class imbalance problems.

## Figures and Tables

**Table 1:** Synthetic balanced dataset comparison.

Datasets	Drift Type	Attributes	Sizes	Class Values
Agarwal	Abrupt and Gradual	10	50k, 100k, 500k, 1M, 2M, 3M	groupA, groupB
LED	Abrupt and Gradual	8	50k, 100k, 500k, 1M, 2M, 3M	0,1,2,3,4,5,6,7,8,9
Mixed	Abrupt and Gradual	11	50k, 100k, 500k, 1M, 2M, 3M	class1, class2
RandomTree	Abrupt and Gradual	11	50k, 100k, 500k, 1M, 2M, 3M	class1, class2

**Table 2:** Synthetic imbalanced dataset comparison.

Dataset	Imbalance Ratio	Attributes	Sizes	Class Values
Agarwal	groupA: 90% groupB: 10%	10	50k, 100k, 150k	groupA, groupB
Mixed	Class1: 90% Class2: 10%	11	50k, 100k, 150k	class1, class2
RandomTree	Class1: 90% Class2: 10%	11	50k, 100k, 150k	class1, class2

**Table 3:** Prediction error percentage in synthetic imbalanced datasets.

**Dataset**	**Agarwal**			
**Artificial**	**ERDDM**	**RDDM**	**EDDM**	**STEPD**
Ab-50k | Gr-50k	0.386 | 0.0089	0.455 | 0.0095	1.396 | 0.0691	0.4996 | 0.985
Ab-100k | Gr-100k	0.391 | 0.0044	0.456 | 0.0047	1.381 | 0.0345	0.5006 | 0.992
Ab-150k | Gr-150k	0.398 | 0.0029	0.457 | 0.0031	1.371 | 0.0230	0.4998 | 0.995
**Dataset**	**Mixed**			
**Artificial**	**ERDDM**	**RDDM**	**EDDM**	**STEPD**
Ab-50k | Gr-50k	0.386 | 0.0089	0.455 | 0.0095	1.396 | 0.0691	0.4996 | 0.985
Ab-100k | Gr-100k	0.391 | 0.0044	0.456 | 0.0047	1.381 | 0.0345	0.5006 | 0.992
Ab-150k | Gr-150k	0.398 | 0.0029	0.457 | 0.0031	1.371 | 0.0230	0.4998 | 0.995
**Dataset**	**RandomTree**			
**Artificial**	**ERDDM**	**RDDM**	**EDDM**	**STEPD**
Ab-50k | Gr-50k	0.386 | 0.0089	0.455 | 0.0095	1.396 | 0.0691	0.4996 | 0.985
Ab-100k | Gr-100k	0.391 | 0.0044	0.456 | 0.0047	1.381 | 0.0345	0.5006 | 0.992
Ab-150k | Gr-150k	0.398 | 0.0029	0.457 | 0.0031	1.371 | 0.0230	0.4998 | 0.995

**Table 4:** Average prediction error in synthetic balanced datasets.

**Dataset**	**Agarwal**				**LED**			
**Artificial**	**ERDDM**	**RDDM**	**EDDM**	**STEPD**	**ERDDM**	**RDDM**	**EDDM**	**STEPD**
Abr-50k	0.3863	0.4552	1.39	0.4996	0.386	0.455	1.40	0.499
Abr-100k	0.3915	0.455	1.38	0.501	0.391	0.4554	1.38	0.506
Abr-500k	0.3909	0.456	1.378	0.4997	0.3909	0.456	1.37	0.49
Abr-1M	0.3901	0.456	1.381	0.499	0.3901	0.456	1.38	0.499
Abr-2M	0.391	0.455	1.386	0.4998	0.391	0.455	1.38	0.49
Abr-3M	0.391	0.456	1.385	0.50	0.391	0.456	1.38	0.51
Gr-50k	0.0089	0.0095	0.069	0.985	0.0089	0.0095	0.069	0.985
Gr-100k	0.0044	0.0047	0.034	0.992	0.0045	0.0047	0.034	0.992
Gr-500k	0.000089	0.000095	0.0069	0.9985	0.000089	0.000095	0.0069	0.9985
Gr-1M	0.000088	0.0000475	0.00345	0.9991	0.000088	0.0000475	0.0035	0.9991
Gr-2M	0.0000224	0.0000237	0.00172	0.9996	0.0000224	0.0000237	0.0017	0.9996
Gr-3M	0.0000149	0.0000158	0.00115	0.9999	0.0000149	0.0000158	0.0012	0.9999
**Dataset**	**RandomTree**				**Mixed**			
**Artificial**	**ERDDM**	**RDDM**	**EDDM**	**STEPD**	**ERDDM**	**RDDM**	**EDDM**	**STEPD**
Abr-50k	0.386	0.455	1.39	0.4996	0.386	0.455	1.39	0.4996
Abr-100k	0.391	0.455	1.38	0.501	0.391	0.4554	1.38	0.501
Abr-500k	0.3909	0.456	1.378	0.4997	0.3909	0.456	1.378	0.499
Abr-1M	0.3901	0.456	1.381	0.499	0.3901	0.456	1.383	0.499
Abr-2M	0.391	0.455	1.386	0.4998	0.391	0.455	1.387	0.49
Abr-3M	0.391	0.456	1.385	0.50	0.391	0.456	1.389	0.51
Gr-50k	0.0089	0.0095	0.069	0.985	0.0089	0.0095	0.069	0.985
Gr-100k	0.0044	0.0047	0.034	0.992	0.0045	0.0047	0.034	0.992
Gr-500k	0.000089	0.000095	0.0069	0.9985	0.000089	0.000095	0.0069	0.9985
Gr-1M	0.000088	0.0000475	0.00345	0.9991	0.000088	0.0000475	0.0035	0.9991
Gr-2M	0.0000224	0.0000237	0.00172	0.9996	0.0000224	0.0000237	0.0017	0.9996
Gr-3M	0.0000149	0.0000158	0.00115	0.9999	0.0000149	0.0000158	0.0012	0.9999

**Table 5:** Mean evaluation time of synthetic imbalanced datasets.

**Dataset**	**Agarwal**			
**Artificial**	**ERDDM**	**RDDM**	**EDDM**	**STEPD**
Ab-50k | Gr-50k	1.82 | 1.56	1.83 | 1.62	1.50 | 1.57	1.50 | 1.36
Ab-100k | Gr-100k	3.08 | 2.71	3.34 | 3.12	3.06 | 3.14	3.01 | 2.88
Ab-150k | Gr-150k	4.54 | 4.72	5.08 | 4.87	4.54 | 4.84	4.72 | 4.25
**Dataset**	**Mixed**			
**Artificial**	**ERDDM**	**RDDM**	**EDDM**	**STEPD**
Ab-50k | Gr-50k	1.53 | 1.63	1.80 | 1.68	1.53 | 1.56	1.57 | 1.39
Ab-100k | Gr-100k	3.02 | 3.00	3.42 | 3.23	3.46 | 3.58	3.87 | 3.26
Ab-150k | Gr-150k	4.26 | 4.85	5.24 | 5.05	4.96 | 4.77	5.45 | 4.99
**Dataset**	**RandomTree**			
**Artificial**	**ERDDM**	**RDDM**	**EDDM**	**STEPD**
Ab-50k | Gr-50k	1.50 | 1.52	1.79 | 1.69	1.59 | 1.63	1.65 | 1.37
Ab-100k | Gr-100k	3.00 | 3.15	3.51 | 3.51	3.78 | 3.75	4.51 | 4.01
Ab-150k | Gr-150k	4.38 | 4.49	5.18 | 5.07	5.56 | 5.21	5.88 | 5.33

**Table 6:** Mean evaluation time of synthetic balanced datasets.

**Dataset**	**Agarwal**				**LED**			
**Artificial**	**ERDDM**	**RDDM**	**EDDM**	**STEPD**	**ERDDM**	**RDDM**	**EDDM**	**STEPD**
Abr-50k	1.48	2.63	1.40	1.45	1.8	2.61	1.28	1.5
Abr-100k	3.10	3.18	2.75	2.71	3.02	3.41	2.72	2.6
Abr-500k	16.41	18.91	17.73	15.61	15.52	17.04	14.21	13.34
Abr-1M	34.22	37.75	31.84	41.18	32.83	34.65	28.97	28.74
Abr-2M	99.13	78.03	81.24	70.22	96.12	74.41	69.36	74.34
Abr-3M	130.21	128.62	133.63	98.96	125.48	121.69	128.67	115.8
Gr-50k	1.76	2.31	1.67	1.70	1.96	1.78	1.51	1.53
Gr-100k	2.90	3.48	3.34	3.36	3.12	3.12	3.54	3.12
Gr-500k	14.74	16.96	15.55	18.39	15.18	16.32	15.23	18.01
Gr-1M	31.41	33.59	30.80	43.60	31.76	32.49	33.99	44.34
Gr-2M	62.99	69.28	59.43	153.7	63.77	69.88	60.65	155.69
Gr-3M	91.94	103.58	91.86	176.25	93.45	104.19	91.22	177.43
**Dataset**	**RandomTree**				**Mixed**			
**Artificial**	**ERDDM**	**RDDM**	**EDDM**	**STEPD**	**ERDDM**	**RDDM**	**EDDM**	**STEPD**
Abr-50k	1.84	2.28	1.73	1.65	1.49	1.71	1.53	1.50
Abr-100k	3.08	3.43	2.69	2.79	2.56	3.32	3.00	3.01
Abr-500k	16.64	18.54	17.39	15.99	14.62	18.24	15.72	15.79
Abr-1M	33.89	37.89	32.48	42.12	30.69	33.75	28.33	31.17
Abr-2M	98.57	80.41	82.38	71.52	93.25	73.91	70.51	76.87
Abr-3M	129.37	131.72	135.51	99.56	121.42	120.44	129.10	118.98
Gr-50k	1.90	2.31	1.67	1.70	1.96	1.78	1.51	1.53
Gr-100k	2.90	3.48	3.34	3.36	3.12	3.12	3.54	3.12
Gr-500k	14.74	16.96	15.55	18.39	15.18	16.32	15.23	18.01
Gr-1M	31.41	33.59	30.80	43.60	31.76	32.49	33.99	44.34
Gr-2M	62.99	69.28	59.43	153.7	63.77	69.88	60.65	155.69
Gr-3M	91.94	103.58	91.86	176.25	93.45	104.19	91.22	177.43

**Table 7:** Average detection delay in synthetic imbalanced datasets.

**Dataset**	**Agarwal**			
**Artificial**	**ERDDM**	**RDDM**	**EDDM**	**STEPD**
Ab-50k | Gr-50k	64.4 | 14	50.2 | 119	48.5 | 76	15.85 | 243
Ab-100k | Gr-100k	65.36 | 14	49.47 | 119	49.64 | 76	14.72 | 243
Ab-150k | Gr-150k	65.89 | 14	50.03 | 119	49.70 | 76	14.61 | 243
**Dataset**	**Mixed**			
**Artificial**	**ERDDM**	**RDDM**	**EDDM**	**STEPD**
Ab-50k | Gr-50k	64.4 | 14	50.2 | 119	48.5 | 76	15.85 | 243
Ab-100k | Gr-100k	65.36 | 14	49.47 | 119	49.64 | 76	14.72 | 243
Ab-150k | Gr-150k	65.89 | 14	50.03 | 119	49.70 | 76	14.61 | 243
**Dataset**	**RandomTree**			
**Artificial**	**ERDDM**	**RDDM**	**EDDM**	**STEPD**
Ab-50k | Gr-50k	64.4 | 14	50.2 | 119	48.5 | 76	15.85 | 243
Ab-100k | Gr-100k	65.36 | 14	49.47 | 119	49.64 | 76	14.72 | 243
Ab-150k | Gr-150k	65.89 | 14	50.03 | 119	49.70 | 76	14.61 | 243

**Table 8:** Average detection delay in synthetic balanced datasets.

**Dataset**	**Agarwal**				**LED**			
**Artificial**	**ERDDM**	**RDDM**	**EDDM**	**STEPD**	**ERDDM**	**RDDM**	**EDDM**	**STEPD**
Abr-50k	64.4	50.2	48.5	15.85	64.4	50.2	48.5	15.8
Abr-100k	65.36	49.475	49.64	14.72	65.36	49.47	49.6	14.75
Abr-500k	65.8	49.39	49.26	14.81	65.8	49.39	49.3	14.81
Abr-1M	64.4	49.21	49.11	14.91	64.4	49.21	49.1	14.92
Abr-2M	65	49.03	48.85	14.80	65	49.03	48.8	14.80
Abr-3M	65.8	48.97	48.79	14.76	65.8	48.97	48.8	14.75
Gr-50k	14	119	76	243	14	119	76	243
Gr-100k	14	119	76	243	14	119	76	243
Gr-500k	14	119	76	243	14	119	76	243
Gr-1M	14	119	76	243	14	119	76	243
Gr-2M	14	119	76	243	14	119	76	243
Gr-3M	14	119	76	243	14	119	76	243
**Dataset**	**RandomTree**				**Mixed**			
**Artificial**	**ERDDM**	**RDDM**	**EDDM**	**STEPD**	**ERDDM**	**RDDM**	**EDDM**	**STEPD**
Abr-50k	64.4	50.2	48.5	15.85	64.4	50.2	48.5	15.85
Abr-100k	65.4	49.47	49.64	14.72	65.36	49.47	49.64	14.72
Abr-500k	65.8	49.39	49.26	14.81	65.8	49.39	49.26	14.81
Abr-1M	64.4	49.21	49.11	14.91	64.4	49.21	49.1	14.92
Abr-2M	65	49.03	48.85	14.80	65	49.03	48.8	14.80
Abr-3M	65.8	48.97	48.79	14.76	65.8	48.97	48.8	14.75
Gr-50k	14	119	76	243	14	119	76	243
Gr-100k	14	119	76	243	14	119	76	243
Gr-500k	14	119	76	243	14	119	76	243
Gr-1M	14	119	76	243	14	119	76	243
Gr-2M	14	119	76	243	14	119	76	243
Gr-3M	14	119	76	243	14	119	76	243

**Table 9:** Total detected drifts in synthetic imbalanced datasets.

**Dataset**	**Agarwal**			
**Artificial**	**ERDDM**	**RDDM**	**EDDM**	**STEPD**
Ab-50k | Gr-50k	390 | 10	100 | 4	247 | 13	210 | 486
Ab-100k | Gr-100k	781 | 10	201 | 4	463 | 13	421 | 1319
Ab-150k | Gr-150k	1171 | 10	302 | 4	693 | 13	628 | 2152
**Dataset**	**Mixed**			
**Artificial**	**ERDDM**	**RDDM**	**EDDM**	**STEPD**
Ab-50k | Gr-50k	390 | 10	100 | 4	247 | 13	210 | 486
Ab-100k | Gr-100k	781 | 10	201 | 4	463 | 13	421 | 1319
Ab-150k | Gr-150k	1171 | 10	302 | 4	693 | 13	628 | 2152
**Dataset**	**RandomTree**			
**Artificial**	**ERDDM**	**RDDM**	**EDDM**	**STEPD**
Ab-50k | Gr-50k	390 | 10	100 | 4	247 | 13	210 | 486
Ab-100k | Gr-100k	781 | 10	201 | 4	463 | 13	421 | 1319
Ab-150k | Gr-150k	1171 | 10	302 | 4	693 | 13	628 | 2152

**Table 10:** Total detected drifts in synthetic balanced datasets.

**Dataset**	**Agarwal**				**LED**			
**Artificial**	**ERDDM**	**RDDM**	**EDDM**	**STEPD**	**ERDDM**	**RDDM**	**EDDM**	**STEPD**
Abr-50k	390	100	247	210	390	100	247	210
Abr-100k	781	201	463	421	781	201	463	421
Abr-500k	3906	1010	2363	1114	3906	1010	2363	1114
Abr-1M	7812	2022	4731	4165	7812	2022	4731	4165
Abr-2M	15624	4040	9525	8335	15624	4040	9525	8335
Abr-3M	20006	6066	14328	12486	20006	6066	14328	12486
Gr-50k	10	4	13	486	10	4	13	486
Gr-100k	10	4	13	1319	10	4	13	1319
Gr-500k	10	4	13	7986	10	4	13	7986
Gr-1M	10	4	13	16319	10	4	13	16319
Gr-2M	10	4	13	32986	10	4	13	32986
Gr-3M	10	4	13	48318	10	4	13	48318
**Dataset**	**RandomTree**				**Mixed**			
**Artificial**	**ERDDM**	**RDDM**	**EDDM**	**STEPD**	**ERDDM**	**RDDM**	**EDDM**	**STEPD**
Abr-50k	390	100	247	210	390	100	247	210
Abr-100k	781	201	463	421	781	201	463	421
Abr-500k	3906	1010	2363	1114	3906	1010	2363	1114
Abr-1M	7812	2022	4731	4165	7812	2022	4731	4165
Abr-2M	15624	4040	9525	8335	15624	4040	9525	8335
Abr-3M	20006	6066	14328	12486	20006	6066	14328	12486
Gr-50k	10	4	13	486	10	4	13	486
Gr-100k	10	4	13	1319	10	4	13	1319
Gr-500k	10	4	13	7986	10	4	13	7986
Gr-1M	10	4	13	16319	10	4	13	16319
Gr-2M	10	4	13	32986	10	4	13	32986
Gr-3M	10	4	13	48318	10	4	13	48318

**Table 11:** Prediction accuracy and mean evaluation time (seconds) in the electricity dataset.

Classifier	AWE		ARF		OzaBag Adwin		Leverage Bagging	
Drift Detectors	Accuracy	Time	Accuracy	Time	Accuracy	Time	Accuracy	Time
**ERDDM**	53.61	0.45	91.43	82.15	51.9	0.43	52.9	0.44
**RDDM**	51.21	0.45	89.42	82.97	49.6	0.48	51.3	0.49
**EDDM**	41.85	0.39	82.91	180.14	40.62	0.34	39.24	0.39
**STEPD**	43.19	0.39	81.34	184.52	44.35	0.38	44.13	0.41

**Table 12:** Prediction accuracy and mean evaluation time (second) of the intrusion detection dataset.

Classifier	AWE		ARF		OzaBag Adwin		Leverage Bagging	
Drift Detectors	Accuracy	Time	Accuracy	Time	Accuracy	Time	Accuracy	Time
**ERDDM**	86.11	7.58	99.93	367.34	75.98	7.45	70.69	8.12
**RDDM**	84.16	7.19	99.91	318.64	74.31	7.34	69.43	7.83
**EDDM**	71.26	5.97	99.1	1093.62	59.34	6.92	62.71	7.14
**STEPD**	69.19	6.66	99.79	1194.12	61.96	9.29	65.19	11.38
